# Prenatal diagnosis of citrullinemia type 1; seven families with c.1168G > A mutation of Argininosuccinate synthetase 1 gene in Southwest Iran: A case series

**DOI:** 10.18502/ijrm.v20i12.12567

**Published:** 2023-01-09

**Authors:** Maryam Hassanlou, Maryam Abiri, Sirous Zeinali

**Affiliations:** ^1^Farzanegan Campus, Semnan University, Semnan, Iran.; ^2^Department of Medical Genetics, Faculty of Medicine, Iran University of Medical Sciences, Tehran, Iran.; ^3^Shahid Akbarabadi Clinical Research Development Unit, Iran University of Medical Sciences, Tehran, Iran.; ^4^Dr. Zeinali's Medical Genetics Laboratory, Kawsar Human Genetics Research Center, Tehran, Iran.; ^5^Department of Molecular Medicine, Biotechnology Research Center, Pasteur Institute of Iran, Tehran, Iran.

**Keywords:** Argininosuccinate synthetase, Chorionic villus sampling, Point mutation.

## Abstract

**Background:**

Citrullinemia type 1 is an autosomal recessive disease resulting in ammonia accumulation in the blood, and if uncontrolled may progress to coma or death in the early months after birth.

**Cases presentation:**

7 families from Southwest Iran having one or more children in their families or relatives, who died in the early months after birth due to citrullinemia type 1 visited for genetic counseling and prenatal diagnosis. Whole-exome sequencing was performed on peripheral blood specimens and chorionic villus samples. Sanger sequencing confirmed the genetic results. Both parents were identified as carriers for the exon 15 c.1168G > A mutation in each family. The fetus in 6 out of 7 families was homozygote for A substitution on the argininosuccinate synthetase 1 gene.

**Conclusion:**

The presence of a common mutation in the argininosuccinate synthetase 1gene in all affected families of Southwest Iran shows a possible population cluster in this area.

## 1. Introduction

Citrullinemia type 1 is an autosomal recessive rare, life-threatening inherited disease in 1:57,000 births with higher incidences in populations with more frequent consanguinity (1). Citrullinemia type 1 includes a classic neonatal acute form, a late-onset milder form, a form that begins during or after pregnancy, and an asymptomatic form. The underlying biochemical defect is a deficiency of the argininosuccinate synthetase (ASS) enzyme due to a mutation in the *ASS1* gene. ASS is one of the 6 enzymes that have a role in the urea cycle, which removes nitrogen from the body (2). The synthesis of argininosuccinate from citrulline and aspartate is catalyzed by ASS enzyme-the 3 ${}^{\text{rd}}$ step in the urea cycle. The enzyme deficiency is characterized by elevated blood citrulline and ammonia levels, which often results in hyperammonemia coma and early neonatal death in affected children due to cerebral edema and encephalopathy if left untreated. In classic form of Citrullinemia several symptom including vomiting, refusal to eat, progressive lethargy, and signs of increased intracranial pressure are seen in infants. Prompt treatment is needed for prolonged survival. The main treatments for ASS deficiency is a low protein and high-calorie diet supplemented with amino acid, particularly arginine. Severe hyperammonemia could result in neurological damage and can be treated with hemodiafiltration (3, 4). An untreated infant with early-onset citrullinemia type 1 is expected to live only 17 days as the longest reported survival time (5). In a few late-onset forms of the disease, symptomatic hyperammonemia develops during childhood and adulthood (6).

The *ASS1* gene has 64 kb length located in chromosome 9q34.1, with 16 exons encoding 412 amino acids. In the *ASS1* gene, several mutations have been reported, of which p.Gly390Arg, p.Arg363Trp, and p.Gly14Ser are the most pathogenic variants with the early onset and severe phenotype (7). The most common mutation is p.Gly390Arg (c.1168G > A) in exon 15, in multiple ethnic backgrounds including the USA, Canada, Spain, Austria, etc. (8). The residue G390 is conserved in *ASS1* gene sequences and is located in α-helix 12, which is critical for multimerization (9, 10). Only a few citrullinemia type 1 have been reported in the Iranian population (11), and none of the prenatal diagnosis (PND) was reported.

In this study, for the first time, we report the identification of Iranian patients with citrullinemia type I in a limited geographic area and postulate a possible population cluster.

## 2. Cases presentation

**Figure 1:**
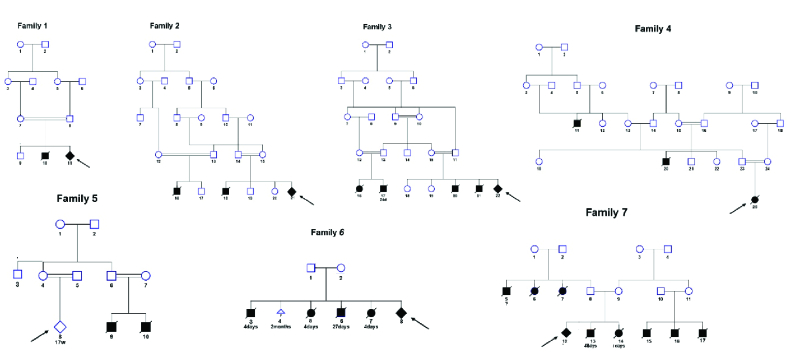
Genetic pedigrees of the 7 families affected with citrullinemia. The arrows indicate probands for PNDs. The colorless square and circle represent the unaffected male and female. Solid colored squares, circles and rhombuses represent the affected people. The rhombus represents individual who died in the early months after the birth with unmentioned sex.

Seven Iranian families from Southwest Iran (2017-2020), having one or more children in their families and/or relatives, who died in the early months after birth due to citrullinemia type 1, were studied (Figure 1). Couples came at 12-14 wk of pregnancy for PND genetic counseling.

Sanger sequencing and haplotyping techniques were performed on couples to confirm their carrier status. To this end, 5 ml of blood was collected from each parent in a heparin / EDTA vial. For chorionic villus sampling from 11-14 wk gestation, duration of pregnancy weeks was confirmed by the gynecologist under ultrasound guidance using a transabdominal approach.

DNA extraction was done on blood and fetal samples by the Genomic DNA Purification Kit (Invitrogen, USA) according to the manufacturer's instructions. Using Nanodrop (Invitrogen) the purity and concentration of extracted DNA were tested.

Whole-exome sequencing was done on all exomes of protein-coding genes and other important genomic regions. Next-generation sequencing was done on Illumina Sequencer to sequence about 100 million reads. Sequencing results were analyzed using international bioinformatics databases and standard software.

For haplotyping, single-nucleotide polymorphisms were selected for areas flanking the* ASS1 *gene (dbSNP, build 151). PCR primers were designed with Primer3. The short tandem repeat typing method was done by gel electrophoresis.

**Figure 2:**
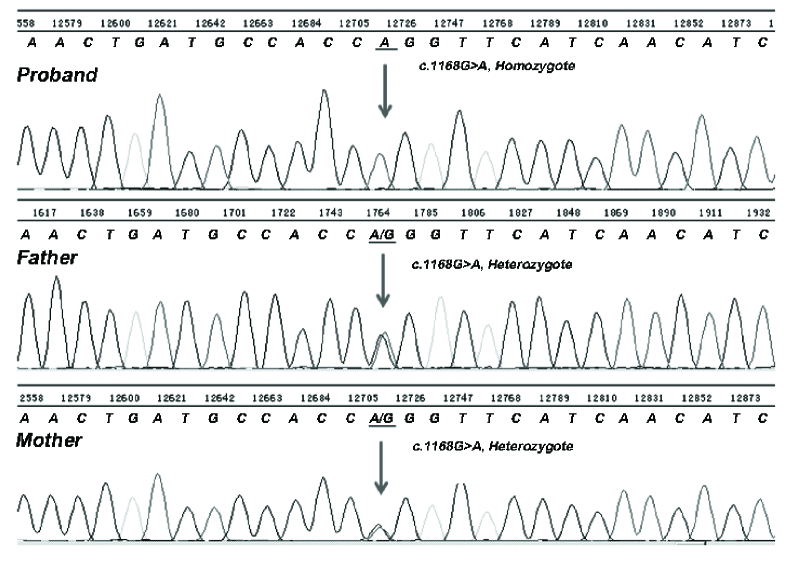
Partial sequencing of *ASS1* gene: The proband has the c.1168G > A mutation, homozygosity. Each parent is a carrier of the mutation. The mutation was not found in normal control.

According to the whole-exome sequencing analysis and haplotyping, all the families except for the 5 ${}^{\text{th}}$ family had a fetus with homozygote missense mutation for *ASS1* in c.1165G > A DNA sequences, which resulted in Gly to Lys substitution (Figure 2).

### Ethical considerations

Ethical approval was obtained from Kowsar Human Genetics Research Center Ethics Committee, Tehran, Iran with the number of 14006319. Written informed consent form was obtained from the child's parents.

## 3. Discussion

In this study, the PND of citrullinemia type 1 was performed in 7 Iranian families from Southwest Iran having one or more children or their relatives children who died in the early months after the birth. Whole-exome sequencing of the coding exons was performed in the parents of the index cases. Haplotype mapping indicated that they were heterozygous for the p.G390R substitution; this mutation was then searched in the fetus. The homozygous substitution was detected in the proband of 6 among 7 families.

The p.Gly390Arg substitution is the most common mutation located in a highly conserved region of exon 15 of the *ASS1* gene. This missense mutation is located in the protein helix and results in the enzymatic inactivation, demonstrating the vitality of this region for multimerization and proper enzyme functioning (12). The homozygous state exclusively results in severely affected patients (13). Other mutations have been found in the *ASS1* gene, but the rate of occurrence is rare (14).

The p.G390R substitution mutation in the *ASS1* gene is a recessive lethal mutation, which means that the inheritance of 2 recessive lethal alleles is fatal. Cystic fibrosis, sickle-cell anemia, and achondroplasia are other examples of human diseases caused by the recessive lethal alleles (15). According to Mendelian inheritance of monogenic disease, following mating between 2 heterozygotes or carriers, 25% of offspring may be recessive for 2 alleles (16-18). In our study, families 6 and 7 had a large number of affected infants. Because citrullinemia type 1 is an autosomal recessive disease and is likely to be inherited from carrier parents by 25% probability in each pregnancy, with current information about this disease, the large number of affected infants in families 6 and 7 is accidental. Further studies are needed to better understand the inheritance behavior of the *ASS1* gene (19).

Our study identified a p.Gly390Arg mutation in *ASS1*, which may be shared among individuals of Southwest Iran and demonstrate a possible population cluster. This information is crucial to identify the carriers in genetic counseling. In addition, this knowledge could increase clinician's awareness in the diagnosis of new cases in a very early neonatal period.

## 4. Conclusion

p.Gly390Arg mutation in *ASS1* shared among individuals of Southwest Iran which demonstrate a possible population cluster.

##  Conflict of Interest

The authors have no conflict of interest.
